# Maritoclax Enhances TRAIL-Induced Apoptosis via CHOP-Mediated Upregulation of DR5 and miR-708-Mediated Downregulation of cFLIP

**DOI:** 10.3390/molecules23113030

**Published:** 2018-11-20

**Authors:** Mi-Yeon Jeon, Kyoung-jin Min, Seon Min Woo, Seung Un Seo, Yung Hyun Choi, Sang Hyun Kim, Dong Eun Kim, Tae-Jin Lee, Shin Kim, Jong-Wook Park, Taeg Kyu Kwon

**Affiliations:** 1 Department of Immunology, School of Medicine, Keimyung University, 2800 Dalgubeoldaero, Dalseo-Gu, Daegu 704-701, Korea; dldkfls2333@naver.com (M.-Y.J.); kyoungjin.min@gmail.com (K.-j.M.); woosm724@gmail.com; (S.M.W.); ssu3885@gmail.com (S.U.S.); god98005@dsmc.or.kr (S.K.); j303nih@dsmc.or.kr (J.-W.P.); 2 Department of Biochemistry, College of Oriental Medicine, Dong-Eui University, Busan 609-735, Korea; choiyh@deu.ac.kr; 3 Department of Pharmacology, School of Medicine, Kyungpook National University, Daegu 702-701, Korea; shkim72@knu.ac.kr; 4 Department of Otolaryngology, School of Medicine, Keimyung University, 2800 Dalgubeoldaero, Dalseo-Gu, Daegu 704-701, Korea; entkde@dsmc.or.kr; 5 Department of Anatomy, College of Medicine, Yeungnam University, Daegu 705-802, Korea; tjlee@med.yu.ac.kr

**Keywords:** maritoclax, TRAIL, cFLIP, DR5, MicroRNA, ER stress

## Abstract

Maritoclax, an active constituent isolated from marine bacteria, has been known to induce Mcl-1 downregulation through proteasomal degradation. In this study, we investigated the sensitizing effect of maritoclax on tumor necrosis factor-related apoptosis-inducing ligand (TRAIL)-induced apoptosis in human renal carcinoma cells. We found that combined treatment with maritoclax and TRAIL markedly induced apoptosis in renal carcinoma (Caki, ACHN and A498), lung cancer (A549) and hepatocellular carcinoma (SK-Hep1) cells. The upregulation of death receptor 5 (DR5) and downregulation of cellular FLICE-inhibitory protein (cFLIP) were involved in maritoclax plus TRAIL-induced apoptosis. Maritoclax-induced DR5 upregulation was regulated by induction of C/EBP homologous protein (CHOP) expression. Interestingly, maritoclax induced cFLIP downregulation through the increased expression of miR-708. Ectopic expression of cFLIP prevented combined maritoclax and TRAIL-induced apoptosis. Taken together, maritoclax sensitized TRAIL-induced apoptosis through CHOP-mediated DR5 upregulation and miR-708-mediated cFLIP downregulation.

## 1. Introduction

The tumor necrosis factor-related apoptosis-inducing ligand (TRAIL) has been considered a promising anticancer agent, because it selectively induces apoptotic cell death in cancer cells, but not in normal cells [1,2,3,4]. There are several reasons why normal cells are more resistant to TRAIL than cancer cells. One of the reasons is that normal cells highly express decoy death receptors (DcRs), which could interfere with the TRAIL-mediated apoptotic pathway [5]. In contrast, since cancer cells have high levels of death receptor (DR)5 proteins, compared with those in normal cells, cancer cells are more sensitive to DR5 receptor agonistic antibodies [6]. In addition, normal cells have redundancy in resistance pathways [7]. Single anti-apoptotic protein inhibition or pro-apoptotic proteins induction is enough to induce TRAIL-mediated apoptosis in cancer cells [8,9]. However, upregulation of DR5 proteins or downregulation of cFLIP fails to increase TRAIL sensitivity in normal astrocytes [10], and inhibition of both cFLIP and XIAP expression or inhibition of both cFLIP expression and activity of anti-apoptotic Bcl-2 proteins increases TRAIL-induced apoptosis in normal fibroblast [7]. Although TRAIL specifically increases cell death in cancer cells, there are many limitations in treatment of TRAIL as an anti-cancer drug. The major limitation of the TRAIL treatment is an acquired resistance to TRAIL [11,12,13,14]. Downregulation of DR expression and upregulation of anti-apoptotic proteins are main cause of acquired TRAIL resistance in cancer cells [15,16,17,18,19]. Combination treatment with TRAIL and chemotherapeutic drugs could effectively reduce TRAIL resistance through modulation of apoptosis-related proteins expression. Therefore, novel TRAIL sensitizing agents are required to overcome TRAIL resistance.

Maritoclax is a natural compound which was identified in marine-derived *Streptomycetes* [20]. It is a novel class of Bcl-2 family inhibitors that selectively induces the proteasomal degradation of Mcl-1 [21]. Recently, it has been reported that maritoclax synergizes with Bcl-xL antagonist ABT-737 to overcome Mcl-1-mediated ABT-737 resistance [22]. Varadarajan et al. reported that maritoclax induces apoptosis in both a Mcl-1-dependent and -independent manner [23]. Maritoclax induces Bax/Bak- and caspase-9-mediated apoptosis in a Mcl-1-dependent manner in NSCLC cell lines, whereas maritoclax also induced apoptosis through increase of mitochondrial fragmentation and ROS accumulation in Mcl-1-deficient MEFs [23]. Furthermore, Eichhorn et al. also reported that maritoclax have equal effectiveness to induce apoptosis in Mcl-1- and Bcl-2-dependent leukemia cells, and fails to induce selective cytotoxicity in Mcl-1-overexpressed Hela cells [24]. Therefore, maritoclax would have other functions to induce apoptosis other than downregulation of Mcl-1 expression.

In this present study, we assess the functional role of maritoclax as a TRAIL sensitizer. Maritoclax does not decrease the expression level of Mcl-1. Maritoclax enhances TRAIL-induced apoptotic cell death through upregulation of DR5 and downregulation of cFLIP expression.

## 2. Results

### 2.1. Effect of Maritoclax on TRAIL-Mediated Apoptosis in Human Renal Carcinoma Caki Cells

**Figure 1:**
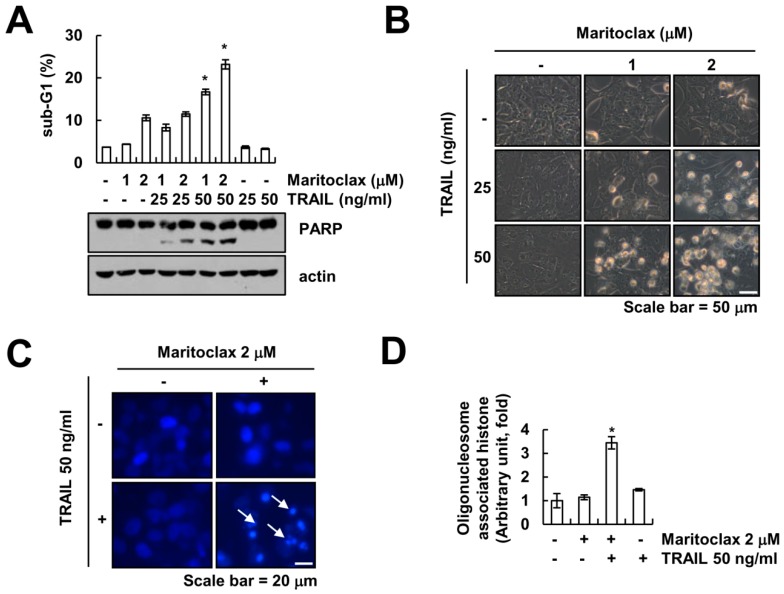
Maritoclax enhances TRAIL-induced apoptosis in Caki cells. (**A**,**B**) Caki cells were treated with TRAIL (25 and 50 ng/mL) in the presence or absence of the indicated concentrations with maritoclax for 24 h. Apoptosis was analyzed as a sub-G1 population by flow cytometer. The protein levels of PARP and actin were determined by Western blotting (**A**). The cell morphology was examined using interference light microscopy (**B**). (**C**,**D**) Caki cells were treated with 50 ng/mL TRAIL in the presence or absence of 2 μM maritoclax for 24 h. The condensation and fragmentation of the nuclei were detected by 4′,6′-diamidino-2-phenylindole staining (**C**). The DNA fragmentation detection kit determined the fragmented DNA (**D**). −, no treatment; +, treatment. The values in graph (**A**,**D**) represent the mean ± SD from three independent samples. * *p* < 0.01 compared to the control by ANOVA and Student-Newman-Keuls methods.

We investigated whether maritoclax, a Mcl-1 specific inhibitor, could sensitize TRAIL-induced apoptotic cell death in Caki cells. Cells were treated with maritoclax alone (1, 2 μM), TRAIL alone (25, 50 ng/mL), and combined maritoclax and TRAIL treatment. As shown in Figure 1A, combined treatment with 2 μM maritoclax plus 50 ng/mL TRAIL markedly induced accumulation of sub-G1 population and cleavage of poly (ADP-ribose) polymerase (PARP). The single treatment with maritoclax or TRAIL did not alter the morphologies, but combined treatment indicated typical apoptotic morphologies in Caki cells (Figure 1B). Furthermore, maritoclax plus TRAIL induced the nuclear condensation and the DNA fragmentation, which is the typical property of apoptosis (Figure 1C,D). These data suggest that maritoclax enhances sensitivity for TRAIL-induced apoptosis.

### 2.2. The Effect of Caspase Activation on Maritoclax Plus TRAIL-Induced Apoptosis

**Figure 2:**
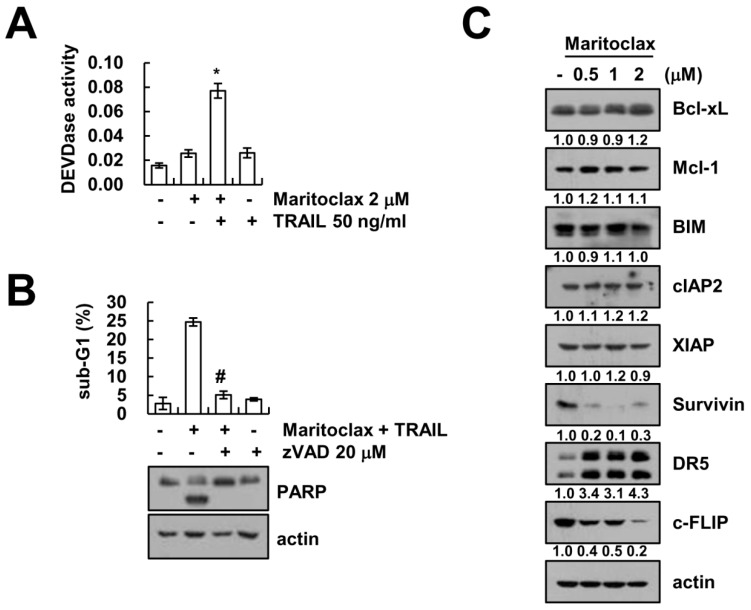
Combined maritoclax and TRAIL treatment induces caspase-dependent apoptosis. (**A**) Caki cells were treated with 50 ng/mL TRAIL in the presence or absence of 2 μM maritoclax for 24 h. Caspase activities were determined using caspase-3 DEVDase assay kits. (**B**) Caki cells were treated with 2 μM maritoclax plus 50 ng/mL TRAIL for 24 h in the presence or absence of 20 μM z-VAD-fmk (zVAD). The sub-G1 fraction was measured by flow cytometry. The protein expression levels were determined by Western blotting. (**C**) Caki cells were treated with indicated concentrations of maritoclax for 24 h. The protein expression levels were determined by Western blotting. The band intensity of the proteins was measured using ImageJ (public domain JAVA image-processing program; http://rsb.info.nih.gov/ij). −, no treatment; +, treatment. The values in graph (**A**,**B**) represent the mean ± SD from three independent samples. * *p* < 0.01 compared to the control. # *p* < 0.01 compared to maritoclax plus TRAIL by ANOVA and Student-Newman-Keuls methods.

Combined treatment with maritoclax and TRAIL induced DEVDase (caspase-3) activation, but not in each single treatment (Figure 2A). Next, we investigated whether caspase activation is involved in maritoclax plus TRAIL-induced apoptosis. Pan-caspase inhibitor, z-VAD-fmk, completely inhibited maritoclax plus TRAIL-induced apoptosis and cleavage of PARP (Figure 2B). To understand the mechanisms behind the induced apoptosis observed in renal carcinoma Caki cells, we analyzed the expression levels of apoptosis-related proteins. Maritoclax induced downregulation of survivin and cFLIP expression, whereas other proteins (Bcl-xL, Mcl-1, Bim, cIAP2, and XIAP) did not change (Figure 2C). In addition, maritoclax markedly upregulated DR5 expression levels (Figure 2C). These data suggest that maritoclax plus TRAIL-induced apoptosis is associated with caspase-dependent pathway, and maritoclax induces downregulation of cFLIP and upregulation of DR5 expression.

### 2.3. The Effect of DR5 Upregulation on Maritoclax Plus TRAIL-Induced Apoptosis

**Figure 3:**
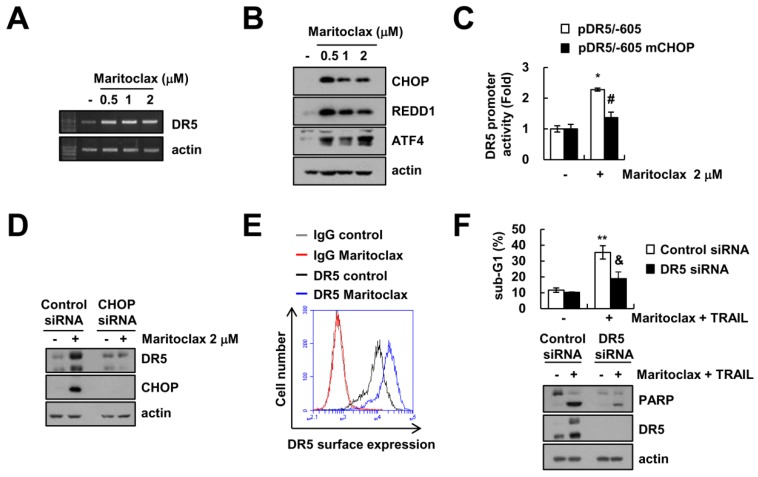
Maritoclax induces CHOP-mediated DR5 upregulation in Caki cells. (**A**,**B**) Caki cells were treated with indicated concentrations of maritoclax for 24 h (**A**) or 9 h (**B**). The mRNA level of DR5 was determined by RT-PCR (**A**). The protein expressions were determined by Western blotting (**B**). (**C**) Caki cells were transiently transfected with a plasmid harboring the luciferase gene under the control of the DR5/-605 and DR5/-605 mutant CHOP (mCHOP) promoter. After transfection, cells were treated with 2 μM maritoclax for 24 h, and the luciferase activity was analyzed. (**D**) Caki cells were transfected with control or CHOP siRNA, and then cells were treated with 2 μM maritoclax for 24 h. The protein expression levels were determined by Western blotting. (**E**) Caki cells were treated with 2 μM maritoclax for 24 h. The cell surface expression level of DR5 was measured by flow cytometry analysis. (**F**) Caki cells were transiently transfected control siRNA or DR5 siRNA, and then cells were treated with 50 ng/mL TRAIL plus 2 μM maritoclax for 24 h. Apoptosis was analyzed as a sub-G1 population by flow cytometry. The protein levels were determined by Western blotting. −, no treatment; +, treatment. The values in graph (**C**,**F**) represent the mean ± SD from three independent samples. * *p* < 0.01 compared to the control. # *p* < 0.01 compared to 2 μM maritoclax-treated pDR5/-605. ** *p* < 0.01 compared to the control & *p* < 0.01 compared to the maritoclax plus TRAIL-treated control siRNA by ANOVA and Student-Newman-Keuls methods.

As shown in Figure 2C, maritoclax upregulated DR5 protein expression. Next, we examined whether maritoclax could increase DR5 expression at a transcriptional level. Maritoclax treatment markedly increased DR5 mRNA levels in a dose-dependent manner (Figure 3A). Several transcription factors such as p53, NF-κB and CHOP/GADD153 have been reported to regulate DR5 expression in various stimuli [25,26,27,28]. Since CHOP is a main transcription factor in endoplasmic reticulum (ER) stress responses [29], we next examined whether maritoclax could induce ER stress. Maritoclax increased the expression of ER stress marker proteins (ATF4, REDD1, and CHOP) (Figure 3B). To determine whether the CHOP is directly associated with maritoclax-induced transcriptional activation of DR5, we employed mutant DR5 promoter, which had lost the ability to interact with CHOP. The promoter activity of DR5 increased with maritoclax treatment, but not in CHOP-mutated DR5 promoter (Figure 3C). We further examined the role of CHOP in maritoclax-mediated DR5 upregulation using small interfering RNA (siRNA). Knockdown of CHOP by siRNA significantly inhibited maritoclax-induced expression of DR5 (Figure 3D). In addition, maritoclax enhanced the surface expression of DR5 (Figure 3E). Downregulation of DR5 by siRNA markedly blocked maritoclax plus TRAIL-induced apoptosis and PARP cleavage (Figure 3F). Taken together, our results clearly indicate that maritoclax-mediated CHOP induction plays a critical role in upregulation of DR5 expression that leads to sensitize TRAIL-induced apoptosis.

### 2.4. The Effect of cFLIP Downregulation on Maritoclax Plus TRAIL-Induced Apoptosis

**Figure 4:**
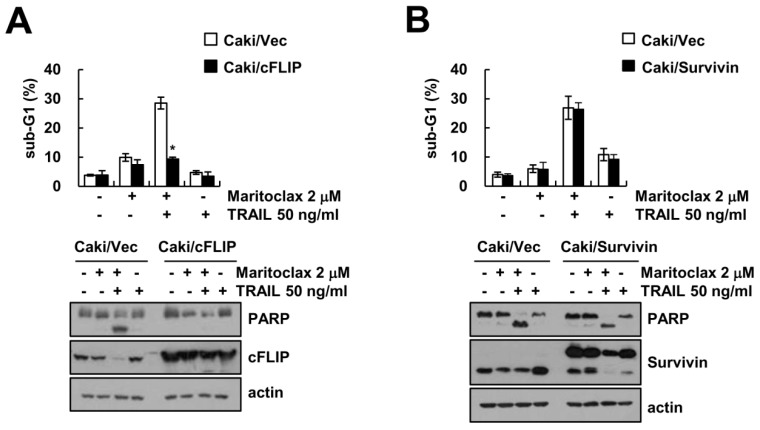
The ectopic expression of cFLIP inhibits maritoclax plus TRAIL-induced apoptosis. (**A**,**B**) Vector cells (Caki/Vec), cFLIP overexpressed cells (Caki/cFLIP) and survivin overexpressed cells (Caki/Survivin) were treated with 50 ng/mL TRAIL in the presence or absence of 2 μM maritoclax for 24 h. The sub-G1 fraction was measured by flow cytometry. The protein expression levels were determined by Western blotting. −, no treatment; +, treatment. The values in graph (**A**,**B**) represent the mean ± SD from three independent samples. * *p* < 0.05 compared to the maritoclax plus TRAIL-treated Caki/Vec by ANOVA and Student-Newman-Keuls methods.

To investigate the role of the downregulation of cFLIP and survivin protein in maritoclax plus TRAIL-induced apoptosis, we used overexpressing cells. Ectopic expression of cFLIP inhibited combined maritoclax and TRAIL treatment-induced apoptosis and PARP cleavage (Figure 4A). However, combined treatment-induced apoptosis in survivin overexpressed cells showed the similar to that of Caki/vector cells (Figure 4B). These data suggest that the maritoclax-induced cFLIP downregulation is indeed essential for combined treatment-induced apoptotic cell death.

**Figure 5:**
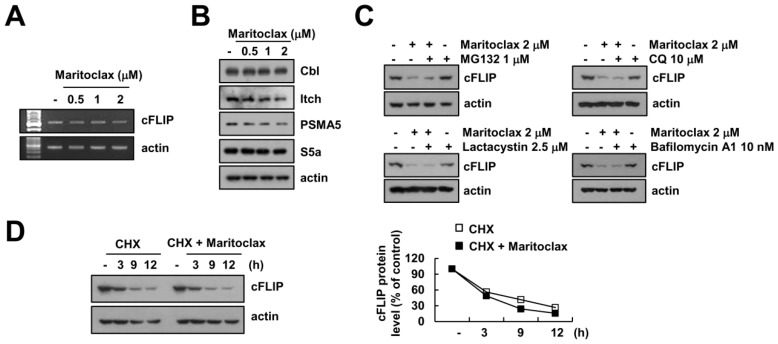
Maritoclax-induced cFLIP downregulation is not associated with proteasomal pathway. (**A**,**B**) Caki cells were treated with indicated concentrations of maritoclax for 24 h. The mRNA level was determined by RT-PCR (**A**). The protein expression levels were determined by Western blotting (**B**). (**C**) Caki cells were pretreated with proteasome inhibitors (1 μM MG132 and 2.5 μM lactacystin) and lysosomal inhibitors (10 μM chloroquine (CQ) and 10 nM bafilomycin A1) for 30 min, and then treated with 2 μM maritoclax for 24 h. The protein expression levels were determined by Western blotting. (**D**) Caki cells were treated with or without 2 μM maritoclax in the presence of 20 μg/mL cycloheximide (CHX) for the indicated time periods. The protein levels were determined by Western blotting (left panel). The band intensity of the cFLIP protein was measured using ImageJ (public domain JAVA image-processing program; http://rsb.info.nih.gov/ij, right panel). −, no treatment; +, treatment.

To clarify the underlying mechanism in maritoclax-induced downregulation of cFLIP, we examined mRNA levels of cFLIP in maritoclax-treated cells. Since maritoclax did not alter cFLIP mRNA expression (Figure 5A), we investigated whether maritolax decreases cFLIP protein expression at post-translation level. Degradation of cFLIP protein is regulated by E3 ligases, Cbl and Itch [30,31]. However, maritoclax did not alter expression levels of Cbl and Itch (Figure 5B). Moreover, expression levels of two critical proteasome subunits, 26S proteasome non-ATPase regulatory subunit 4 (PSMD4/S5a) and 20S proteasome subunit alpha type 5 (PSMA5), were not changed by maritoclax (Figure 5B). To further confirm the involvement of proteasome or lysosome on maritoclax-induced downregulation of cFLIP expression, we used proteasome inhibitors (MG132 and lactacystin) and lysosomal inhibitors (chloroquine and bafilomycin A1). Maritoclax-induced cFLIP down-regulation was not changed by both inhibitors (Figure 5C).

Next, we examined the effect of maritoclax on the protein stability of cFLIP using protein biosynthesis inhibitor, cycloheximide (CHX). Combined treatment with CHX plus maritoclax showed similar degradation pattern to CHX alone treatment (Figure 5D). Therefore, our data suggested that downregulation of cFLIP is independent of degradation by proteasome and lysosome.

**Figure 6:**
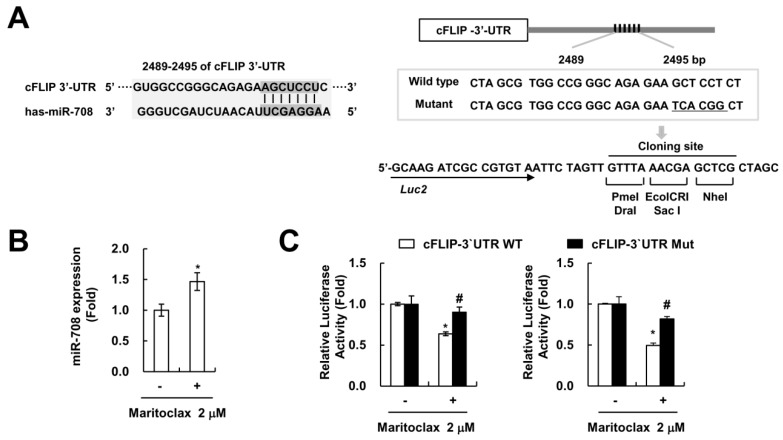
Maritoclax induces miR-708 mediated cFLIP downregulation. (**A**) miR-708 binding sites in the 3′-UTR of cFLIP mRNA (left panel). Schematic representation of putative miR-708 target sites in cFLIP 3′-UTR, and the mutated nucleotides in cFLIP 3′-UTR mutant (right panel). (**B**) Caki cells were treated with 2 μM maritoclax for 24 h. The miR-708 expression levels were analyzed by qPCR. (**C**) Luciferase constructs for cFLIP 3′-UTR wild-type (WT) and cFLIP 3′-UTR mutant (Mut) were transfected and then further culture for 24 h. Caki (left panel) and A549 (right panel) cells were treated with 2 μM maritoclax for 24 h, and the luciferase activity was analyzed. −, no treatment; +, treatment. The values in graph (**B**,**C**) represent the mean ± SD from three independent samples. * *p* < 0.05 compared to the control. # *p* < 0.05 compared to the maritoclax-treated cFLIP 3′-UTR WT by ANOVA and Student-Newman-Keuls methods.

miR-708 is known to target cFLIP and increases TRAIL sensitivity in renal carcinoma cells [32]. We previously reported that cFLIP 3′-UTR contains potential binding site for miR-708 at nucleotides 2489 to 2495 (Figure 6A). Maritoclax treatment induced miR-708 expression (Figure 6B). We constructed luciferase system for miR-708 binding sequence of cFLIP wild type and mutant type (Figure 6A). Maritoclax markedly suppressed the luciferase activity of 3′-UTR in cFLIP (Figure 6C). In contrast, the luciferase activity of the reporter vector containing a mutant 3′-UTR in cFLIP was not altered by maritoclax treatment in human renal carcinoma Caki and human lung carcinoma A549 cells (Figure 6C). Thus, these data indicate that induction of miR-708 by maritoclax have a role in downregulation of cFLIP.

### 2.5. Effect of Combined Treatment with Maritoclax and TRAIL on Apoptosis in Other Cancer Cells

**Figure 7:**
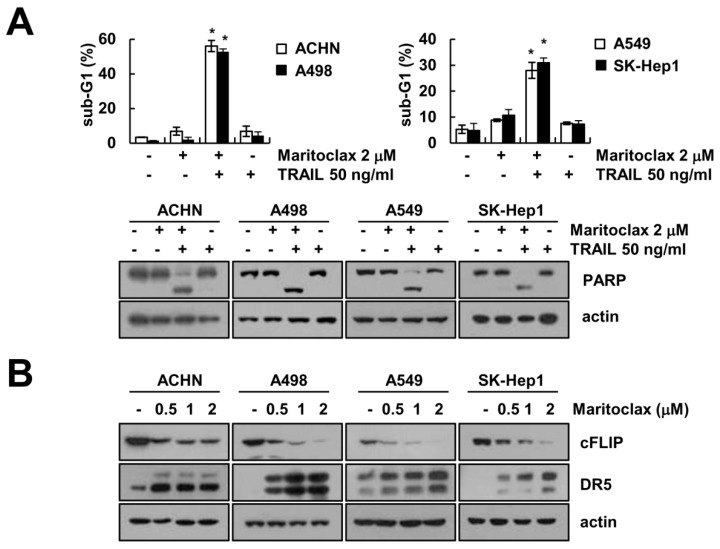
The effects of combined treatment with maritoclax and TRAIL on apoptosis in other carcinoma cells. (**A**) ACHN and A498 (renal carcinoma), A549 (lung carcinoma) and SK-Hep1 (hepatocellular carcinoma) cells were treated with 50 ng/mL TRAIL in the presence or absence of 2 μM maritoclax for 24 h. The level of apoptosis was measured by the sub-G1 fraction using flow cytometry. The protein expression levels were determined by Western blotting. (**B**) Cells were treated with the indicated concentrations of maritoclax for 24 h. The protein expression levels were determined by Western blotting. −, no treatment; +, treatment. The values in graph (**A**) represent the mean ± SD from three independent samples. * *p* < 0.05 compared to the control by ANOVA and Student-Newman-Keuls methods.

Next, we investigated that the effect of maritoclax on TRAIL-induced apoptosis in other renal carcinoma (ACHN and A498) and other type carcinoma (lung carcinoma A549 and hepatocellular carcinoma SK-Hep1). As shown in Figure 7A, combined maritoclax and TRAIL treatment markedly enhanced the sub-G1 population and PARP cleavage in all tested cells. Maritoclax induced downregulation of cFLIP and upregulation of DR5 in dose-dependent manner in cancer cells (Figure 7B). These data indicate that maritoclax enhances TRAIL-mediated apoptosis in various cancer cells.

## 3. Discussion

Our results demonstrated that combined maritoclax and TRAIL treatment induced apoptosis through upregulation of DR5 expression and downregulation of cFLIP. Maritoclax-induced DR5 upregulation was caused by the induction of CHOP which is an ER stress master transcription factor. Maritoclax upregulated miR-708 expression, and then suppressed cFLIP expression. Downregulation of DR5 and ectopic expression of c-FLIP blocked apoptosis in combined maritoclax and TRAIL-treated cells. Therefore, our data suggest that maritoclax could be an effective TRAIL sensitizer through modulation of DR5 and cFLIP expression in cancer cells.

Maritoclax is identified as a Mcl-1 specific inhibitor, and inhibitory mechanism is related with degradation of Mcl-1 by proteasome [21]. Since melanoma has high levels of Mcl-1 expression, these cells have resistance to ABT-737 (a selective inhibitor of Bcl-2, Bcl-xL, and Bcl-w)-induced apoptosis. Combined treatment with maritoclax and ABT-737 enhances apoptosis through downregulation of Mcl-1 expression in melanoma cells [33]. Doi et al. also reported that maritoclax could kill acute myeloid leukemia cells and primary cells through proteasomal degradation-mediated Mcl-1 downregulation [22]. However, maritoclax (2 μM) did not induce Mcl-1 downregulation in Caki cells (Figure 2C). Previous studies reported that effect of maritoclax on downregulation of Mcl-1 is dependent on cell types [23,24]. This discrepancy might be due to different cancer cell contexts and expression levels of ubiquitin-proteasome system. In our study, one of mechanisms of maritoclax-mediated TRAIL sensitization is upregulation of DR5 expression. As shown in Figure 3F, knockdown of DR5 by siRNA markedly blocked maritoclax plus TRAIL-induced apoptosis. Multiple studies have been investigated the transcriptional regulation of DR5 by a variety of transcription factors such as p53, CHOP, NF-κB, Sp1, FOXO, and YY1 [34]. We found that maritoclax induced ER stress marker proteins, such as CHOP, REDD1, and ATF4 (Figure 3B). Therefore, we tested the involvement of CHOP on maritoclax-induced DR5 expression. Maritoclax failed increase of CHOP-mutated DR5 promoter activity (Figure 3C), and CHOP knockdown by siRNA markedly suppressed maritoclax-induced expression of DR5 (Figure 3D). These findings suggest that maritoclax-induced ER stress has a critical role in CHOP-dependent DR5 expression to enhance TRAIL-induced apoptosis.

The other mechanism of maritoclax-mediated TRAIL sensitization is down-regulation of cFLIP expression (Figure 4A). Interestingly, maritoclax-mediated cFLIP degradation was not associated with ubiquitin-proteasome pathway and/or autophagy-lysosome pathway. As shown in Figure 5C, inhibitors of proteasome and lysosome did not abolish maritoclax-mediated cFLIP degradation. In addition, maritoclax did not change protein stability of cFLIP (Figure 5D). It has been known that microRNAs (miRNA) negatively regulate the expression of multiple genes by posttranscriptional repression [35]. The expression levels of cFLIP are regulated by a variety of miRNA such as miR-512-3p [36], miR-1246, miR-320a, and miR-196b-5p [37]. Recently, we reported that miR-708 negatively regulated cFLIP expression by binding to the 3′-UTR of cFLIP, resulted in induction of TRAIL sensitivity [32]. Maritoclax upregulated miR-708 expression and did not activate luciferase activity in mutant 3′-UTR in cFLIP (Figure 6B,C). Our data suggest that maritoclax induced cFLIP downregulation through upregulation of miR-708. However, further studies are required to examine other miRNA are involved in maritoclax-induced cFLIP suppression. Functional studies on miR-708 in renal cell carcinoma (RCC) showed that miR-708 was identified as target for survivin, Zinc finger E-box-binding homeobox 2 (ZEB2) and B lymphoma Mo-MLV insertion region 1 homolog (BMI1), and expression level of miR-708 was widely reduced in RCC patients [38]. ZEB2 acts as a transcriptional repressor of E-cadherin and increases invasion [39], and high levels of BMI1 is correlated with EMT characteristics in cancer cells [40]. Therefore, maritoclax could have multiple anti-cancer activities through miR-708-mediated modulation of anti-apoptotic proteins expression and EMT.

In conclusion, maritoclax enhances TRAIL-mediated apoptosis through the regulation of DR5 and cFLIP expression in the human renal carcinoma cells. Therefore, maritoclax may be an attractive sensitizer for TRAIL resistance cancer cells.

## 4. Materials and Methods

### 4.1. Cell Cultures and Materials

Human renal carcinoma cells (Caki, ACHN and A498), human lung cancer cells (A549), and human hepatocellular carcinoma cells (SK-Hep1) were obtained from the American Type Culture Collection (Manassas, VA, USA). The culture medium used throughout these experiments was Dulbecco’s modified Eagle’s medium (DMEM) (Welgene, Gyeongsan, Korea) containing 10% fetal bovine serum (Welgene, Gyeongsan, Korea), 20 mM HEPES buffer (Thermo Fisher Scientific, Waltham, MA, USA) and 100 μg/mL gentamicin (Thermo Fisher Scientific, Waltham, MA, USA). Maritoclax was purchased from Selleckchem (Houston, TX, USA). The z-VAD-fmk, TRAIL and anti-survivin antibody were obtained from R&D system (Minneapolis, MN, USA). Anti-DR5, anti-PARP, anti-S5a and anti-PSMA5 antibodies were obtained from Cell Signaling Technology (Beverly, MA, USA). Anti-Bim and anti-XIAP antibodies were obtained from BD Biosciences (San Jose, CA, USA). Anti-Mcl-1, anti-cIAP2, anti-Bcl-xL, anti-CHOP, anti-ATF4, anti-Cbl and anti-Itch antibodies were obtained from Santa Cruz Biotechnology (Santa Cruz, CA, USA). Anti-cFLIP and anti-REDD1 antibodies were obtained from ALEXIS Corporation (San Diego, CA, USA). Lactacystin was purchased from Biomol Research Laboratories (Plymouth Meeting, PA, USA). MG132 was obtained Calbiochem (San Diego, CA, USA). Anti-actin antibody, chloroquine, bafilomycin A1 and cycloheximide were purchased from Sigma Chemical Co. (St. Louis, MO, USA).

### 4.2. Western Blot Analysis and Flow Cytometry Analysis

Total lysates were obtained using modified RIPA lysis buffer as described previously [41,42,43]. The proteins were separated by SDS-PAGE and transferred onto an Immobilon-P membrane (GE Healthcare Life Science, Pittsburgh, PO, USA). The membranes were exposed using an enhanced chemiluminescence Western blot kit (EMD Millipore, Darmstadt, Germany). For apoptosis analysis, cells were harvested and fixed with 100 % ethanol for 2 h at 4 °C. Then cells were resuspended in 50 μg/mL RNase for 30 min at 37 °C, and added to 50 μg/mL propidium podide. The stained cells were analyzed by flow cytometry (BD Biosciences, San Jose, CA, USA).

### 4.3. Caspase Activity Assay, DNA Fragmentation Assay and DAPI Staining

To evaluate caspase-3 (DEVDase) activity, cell were treated with TRAIL in the presence or absence of maritoclax, and 20 μg of cell lysates were incubated with reaction buffer. For DAPI staining, cells were stained with 300 nM 4′,6′-diamidino-2-phenylindole solution (Roche, Mannheim, Germany), and using the cell death detection ELISA plus kit (Boehringer Mannheim, Indianapolis, IN, USA), we performed DNA fragmentation assay as described in our previous studies [41].

### 4.5. Reverse Transcription-Polymerase Chain Reaction (RT-PCR)

For obtaining of cDNA, total RNA was prepared using the TriZol reagent (Life Technologies, Gaithersburg, MD, USA), and used M-MLV reverse transcriptase (Gibco-BRL, Gaithersburg, MD, USA). For PCR, we used DNA polymerase with primers targeting actin and cFLIP [30]. The amplified products were separated by electrophoresis on a 2% agarose gel and detected under ultraviolet light.

### 4.6. Detection of DR5 on Cell Surface

Detached cells by 0.2 % EDTA (Sigma Chemical Co., St. Louis, MO, USA) were washed with PBS (Thermo Fisher Scientific, Waltham, MA, USA), and then suspended in 100 μM PBS including 10% FCS and 1% sodium azide Sigma Chemical Co., St. Louis, MO, USA), and added primary antibody (DR5-phycoerythrin; Abcam, Cambridge, MA, USA) for 1 h at room temperature. Then, the cells washed with PBS including 10% FCS and 1% sodium azide, and DR5 on cell surface was detected by flow cytometry.

### 4.7. Real-Time Quantitative RT-PCR for Detection of miR-708

Small RNAs were extracted using the miRNEasy RNA Isolation Kit (Qiagen, Germantown, MD, USA), and RT reactions were performed using a TaqMan® MicroRNA Reverse Transcription Kit and TaqMan® MicroRNA assays specific to the mature form of miR-708. Real-time PCR reactions were performed using the TaqMan Universal PCR Master Mix No AmpErase UNG (Applied Biosystems, Foster City, CA, USA) on an ABI PRISM 7000 Sequence Detection System (Applied Biosystems).

### 4.8. Statistical Analysis

The data were analyzed using a one-way ANOVA and post-hoc comparisons (Student-Newman-Keuls) using the Statistical Package for Social Sciences 22.0 software (SPSS Inc., Chicago, IL, USA).

## Abbreviations

TRAIL: Tumor necrosis factor-related apoptosis-inducing ligand; DR5: Death receptor 5; PARP: Poly (ADP-ribose) polymerase; CHOP: C/EBP homologous protein; PSMD4/S5a: 26S proteasome non-ATPase regulatory subunit 4; PSMA5: 20S proteasome subunit alpha type 5; cFLIP: Cellular FADD-like IL-1β-converting enzyme-inhibitory protein; 3′-UTR: 3′-untranslated region.
